# Boron application increases growth of Brazilian Cerrado grasses

**DOI:** 10.1002/ece3.6367

**Published:** 2020-05-29

**Authors:** Luciola Santos Lannes, Harry Olde Venterink, Matheus Roberto Leite, Jaqueline Nascimento Silva, Martina Oberhofer

**Affiliations:** ^1^ Department of Biology and Animal Sciences State University of São Paulo (UNESP) Ilha Solteira Brazil; ^2^ Department of Biology Vrije Universiteit Brussel Brussels Belgium; ^3^ Department of Pharmacognosy University of Vienna Vienna Austria

**Keywords:** growth, limitation, micronutrients, nutrients

## Abstract

Nutrients are known to limit productivity of plant communities around the world. In the Brazilian Cerrado, indirect evidences point to phosphorus as the main limiting nutrient, but some fertilization experiments suggest that one or more micronutrients might play this role. Boron is one of the essential micronutrients for plants. Agronomically, it received some attention, but it has mostly been neglected in ecological studies assessing the effects of nutrients on plant growth. Through field fertilization and mesocosm experiments in a degraded area in the Cerrado, we show that boron addition increased biomass production of herbaceous vegetation. This could be related to a lower aluminum uptake in the boron fertilized plants. Even considering that plant growth was promoted by boron addition due to aluminum toxicity alleviation, this is the first study reporting boron limitation in natural, noncultivated plant communities and also the first report of this kind in vegetative grasses. These results contribute to disentangling patterns of nutrient limitation among plant species of the species‐rich, aluminum‐rich, and nutrient‐poor Cerrado biome and highlight the potential role of micronutrients, such as boron, for growth of noncrop plants. Understanding how nutrient limitation differs among functional groups in the highly biodiverse areas founded on ancient tropical soils may help managing these plant communities in a changing world.

## Graphical abstract

Grasses in the Brazilian Cerrado increase growth under boron supply. These results are new for two reasons: First, it shows that growth of grasses can be limited by boron, and second, that natural plant communities respond to a micronutrient added alone. The mechanism behind the response to boron addition seems to be related to aluminum toxicity alleviation in the highly weathered, aluminum‐rich Cerrado soils.

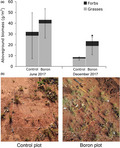

## 1 INTRODUCTION

Nutrients, water, and light are the three main resources that limit plant growth (Craine & Dybzinski, 2013). Among the 17 elements essential for plant growth and development, nitrogen (N) and phosphorus (P) are often mentioned as the ones that most frequently limit natural plant communities (Bobbink et al., 2010; Elser et al., 2007). However, N and P limiting grassland productivity more frequently than other nutrients could be caused by a bias, because these two nutrients are also far more often investigated in fertilization studies than any other nutrient. There are, however, several examples of fertilization experiments in natural ecosystems where another nutrient than N or P, or non‐nutrient resources (water, light, CO_2_), was growth‐limiting for the plant community (e.g., Fay et al., 2015; Harpole et al., 2011). Which other nutrient or resource often remains unknown in these studies, and could be another macro‐ or a micronutrient whose effect on vegetation biomass was not assessed specifically.

The Cerrado is the second largest biome in Brazil, occupying an area of approximately 2 million km^2^ and harboring 12,000 vascular plant species (Mendonça et al., 2008). Cerrado soils are generally P‐poor and contain plants with low P contents (Batmanian & Haridasan, 1985; Villela & Haridasan, 1994), and its plants often have strategies to conserve P, as high P use efficiency and resorption rate (Kozovits et al., 2007; Nardoto, Bustamante, Pinto, & Klink, 2006). These observations have led scientists to suggest that P is the main nutrient limiting plant growth in this biome (Miatto, Wright, & Batalha, 2016; Sousa‐Souto, Schoereder, & Schaefer, 2007). Fertilization experiments at six sites in Cerrado grasslands in the Brazilian Central Plateau, however, showed that only alien invasive plants (*Melinis minutiflora* and *Urochloa decumbens*) are limited by low soil P, whereas growth of native C_4_ grasses was stimulated more by a treatment with cations and micronutrients, either alone or in combination with N and P addition (Lannes, Bustamante, Edwards, & Olde Venterink, 2016). These results challenge the common assumption that Cerrado vegetation is limited by P, suggesting that nutrients other than N or P may limit growth of Cerrado herbaceous vegetation.

The results of Lannes et al. (2016) raise the question of which nutrients may limit the productivity of Cerrado C_4_ grasses. In the mentioned experiment, potassium (K), calcium (Ca), magnesium (Mg), iron (Fe), manganese (Mn), zinc (Zn), boron (B), copper (Cu), and molybdenum (Mo) were added in combination; thus, any of these nutrients could limit or colimit the productivity in that area. In a recent study investigating the effects of sheep dung deposition as possible stimulants of plant growth in a degraded Cerrado area in Sao Paulo, Teixeira, Rezende, and Lannes (2019) detected that this treatment stimulated aboveground biomass growth of native Cerrado grasses. Boron was the only element among 11 determined macro‐ and micronutrients that significantly increased in concentration in soil of plots treated with dung addition. This increase in soil B concentration concomitantly to higher growth of Cerrado grasses stimulates investigation about the effect of B supply upon grassland productivity in the Cerrado.

Boron limits agricultural productivity worldwide at both high and low availabilities (Gupta, 1993). Cerrado soils are B deficient, and its addition can promote yield increase in crop plants (Shorrocks, 1997). Ecologically, however, B has only recently been proposed as a key element for structuring natural tree communities in rainforests in Panama (Steidinger, 2015), but contrasting results were reported from a long‐term experiment in the field (Turner et al., 2017). Yet, no convincing evidences of B limitation in natural plant communities have been reported. Previous studies on the effects of B in B‐limited plants have shown a relationship between B limitation and aluminum (Al) uptake of plants. The elements B and Al have the same trivalent positive charge, so if B supply is not sufficient, Al is absorbed by the plant instead leading to Al poisoning and reduced plant growth. This effect occurs mainly at soil pH between 4.0 and 4.5 (Blevins & Lukaszewski, 1998; Lenoble, Blevins, Sharp, & Cumbie, 1996). It is noteworthy that these effects were observed in forbs, but were not found in wheat, a domesticated C_3_ grass (Taylor & MacFie, 1994).

The Cerrado is a biodiversity hotspot (Myers, Mittermeier, Mittermeier, da Fonseca, & Kent, 2000) intensely degraded because of land use intensification (Klink & Machado, 2005). Therefore, unraveling nutrient limitation patterns in these highly diverse and threatened plant communities is pivotal since vegetations limited by different nutrients require different management practices. The aim of this study was to investigate the effects of B fertilization on biomass production of a herbaceous Cerrado plant community, through a field fertilization experiment performed in the same area of the Teixeira et al. (2019) study. Additionally, the effect of B fertilization on individual plant species (native, alien noninvasive, and alien invasive species) in a mesocosm experiment with Cerrado soil was investigated. We hypothesize that biomass production of the plant community (field study), and of at least some of the individual native Cerrado species (mesocosm study), will be enhanced by B fertilization compared to an unfertilized control.

## 2 MATERIAL AND METHODS

### 2.1 Field fertilization experiment

The field fertilization experiment was designed to study the effect of B application on the growth of the herbaceous vegetation in the Cerrado and was conducted in an area in Central Brazil (20°23.012ʹS 51°23.624W). This region has a humid tropical climate with a pronounced dry season from May to September and a rainy season that concentrates more than 70% of the rainfall from October to April. Soils are mostly composed by acid Latosols. They are well‐drained, with a very profound depth to the bedrock, stained red by a high Fe and Al contents, and are clay‐rich, structurally strong but poor in mineral nutrients (Embrapa, 2018).

Previously, surface soil removal of approximately 8.6 m of the original soil profile for constructing the foundation of the dam of the hydropower plant from Ilha Solteira effectively eliminated the native vegetation of a massive area in this region (Alves, Nascimento, & Souza, 2012; Teixeira et al., 2019). The area was vastly colonized by green algae and bryophytes, but taller plants were still scarce and patchy, which may suggest that nutrients and/or water limitation hamper ecological succession in the studied area.

In a homogeneous grassland of approximately 0.35 ha within this degraded area, 20 plots of 1 square meter each were established with a minimum buffer area of 1 meter between plots in all directions. Vegetation was composed of grasses, forbs, and legumes, covering approximately 60% of the area, of which 90% were dominated by the naturalized C_4_ grass *Hyparrhenia rufa* and the other 10% included the native C_4_ grasses *Sporobolus indicus* and *Setaria parviflora*. In November 2016, 10 plots received 0.02 g/m^2^ of B as Borax (Na_2_B_4_O_7_·10H_2_O) diluted in 2 L of distilled water (“Boron” treatment) and the other ten plots received 2 L of distilled water (“Control” treatment). The distribution of the “Boron” and the “Control” plots followed a completely randomized design. The quantity of B was based on Cech, Kuster, Edwards, and Olde Venterink (2008) and Lannes et al. (2016). After seven months (in June 2017), vegetation of each plot was clipped at 5 cm height from the ground, taken to the laboratory, sorted into two functional groups (“grasses” and “forbs”), dried at 70°C during 72 hr, and then weighed. Directly after clipping the vegetation, B was applied with the same dosage once more. In a second harvest 6 months later (in December 2017), the vegetation was clipped, sorted into functional groups, dried, and weighed again. Samples collected at this occasion were ground, and foliar macro‐ and micronutrients were determined colorimetrically or by means of atomic absorption, after combustion or digestion according to methods of Malavolta, Vitti, and Oliveira (1997); see details per nutrient in Appendix S1.

At the end of the experiment, three top 10‐cm soil cores (5 cm diameter) were randomly collected in each plot and pooled to form a composite sample per plot. The soil was air‐dried to constant weight, sieved, and ground. Soil chemical characteristics were determined according to methods of Raij, Andrade, Cantarella, and Quaggio (2001) as detailed in Appendix S2. Soil extractable B was measured after extraction of 10 cm^−3^ dry soil with 20 ml barium chloride 6 mM solution by heating in a microwave at 490 W for 5 min. The B concentration was measured colorimetrically using the azomethine‐H method and adsorption at 420 nm on a spectrophotometer (Varian 50 Probe). Extractable Ca, Cu, Fe, Mg, Mn, K, and Zn concentrations were measured by means of atomic adsorption, after various chemical extractions (details in Appendix S2). Extractable Al was measured after extraction with 1 M KCl and titration with NaOH using the phenolphthalein method. Extractable P was measured colorimetrically after extraction with ion exchange resin and then washed with 0.8 M NH_4_CL and 0.2 M HCl. Extractable sulfur (S) was measured colorimetrically after extraction with activated charcoal and 0.01M Ca(H_2_PO_4_). Soil pH was measured in a soil–water suspension (10 g dry soil in 50 ml deionized water) using a Metrohm Herisau pH meter with a Mettler Toledo electrode. Soil organic matter content was determined colorimetrically after extraction for 10 min. with 0.667 M sodium dichromate and 5 M sulfuric acid. All soil chemical characteristics were determined according to Raij et al. (2001) as detailed in Appendix S2.

### 2.2 Mesocosm experiment

A mesocosm experiment with eight common Cerrado plants was performed aiming to test plant growth subjected to B addition in a controlled environment. Species comprised the naturalized grasses *Hyparrhenia rufa*, *Digitaria insularis,* and *Melinis repens*; the alien invasive grasses *Melinis minutiflora* and *Urochloa decumbens*; the native legume *Calopogonium mucunoides;* and the native forbs *Waltheria indica* and *Sida cerradoensis* (both Malvaceae). Per mesocosm, three individuals of each species were grown in 20 1‐L pots filled with a 2:1 mixture of Cerrado soil and washed quartz sand. This sand had negligible available nutrients and organic matter contents. Ten pots per species with all three plants alive and healthy were selected for the experiment, and other six unvegetated mesocosms were set up as soil Controls. Half of the mesocosms received 2 mg/kg B as Borax following Galrão (1991), Fageria (2000), and Barman, Shukla, Datta, and Rattan (2014), yielding five replicates per species and three replicates per unvegetated Control.

Soil was collected in November 2018 nearby the field fertilization site (20°23.012ʹS 51°23.624W), and plants were germinated from seeds 8 weeks prior to the beginning of the experiment. The experiment was conducted in a screenhouse at the *campus* of the State University of Sao Paulo—UNESP—in the municipality of Ilha Solteira, approximately 10 km from the field fertilization site. The experiment was performed between 16 November 2018 and 22 January 2019. Each plant was harvested and separated into roots and aboveground parts, and aerial height was measured. Plants were then oven‐dried at 70°C during 72 hr prior to weighing into aerial and root biomass. Aerial and root samples were then mixed and ground to determine foliar nutrients following the same methods as used for the field experiment material.

### 2.3 Data analyses

Standardized differences between means of plant biomass, height, and plant nutrient concentrations between B fertilized and Control plots were calculated as Cohen's *d* effect size measures (Cohen, 1988). Effects of B additions on all plant and soil characteristics were tested through Student's *t* tests (*p* < .05) using Stata IC‐15 (StataCorp, 2017). Data were log‐transformed if necessary to guarantee normal distribution of residues.

## 3 RESULTS

### 3.1 Field fertilization experiment

Soil B concentrations increased significantly in the B fertilized plots, unlike the other measured soil variables (Table 1). Soil chemical characteristics from the field fertilization experimental area (Table 1) showed that pH and available P values increased (though not significantly), respectively, from 4.4 to 4.9 (*p = *.333) and from 1.2 to 1.9 mg/kg (*p = *.106) in plots after fertilization.

**TABLE 1:** Soil characteristics of Control and Boron fertilized plots (0.02 g/m^2^ boron as Borax) in a Cerrado grassland (for the methods used, see Appendix S1)

Soil attribute	Control plots	Boron plots	*t*‐Value	*p*‐Value
Boron (mg/kg)	0.02 (0.01)	0.11 (0.03)	7.612	<.001
Aluminum (mg/kg)	32 (5)	32 (6)	0.098	.923
Calcium (mg/kg)	92 (18)	96 (12)	0.575	.572
Copper (mg/kg)	10 (3)	10 (3)	0.355	.726
Iron (mg/kg)	5.0 (2.1)	4.5 (1.1)	0.394	.698
Magnesium (mg/kg)	31 (7)	32 (11)	0.116	.908
Manganese (mg/kg)	5.6 (1.2)	5.1 (1.1)	0.943	.358
Phosphorus (mg/kg)	1.2 (0.6)	1.9 (1.0)	1.698	.106
Potassium (mg/kg)	27.4 (19)	23.4 (11.7)	0.478	.638
Sulfur (mg/kg)	20.2 (5.9)	16.4 (2.7)	1.881	.076
Zinc (mg/kg)	0.06 (0.02)	0.04 (0.02)	1.658	.114
pH	4.4 (0.7)	4.9 (1.1)	0.993	.333
Organic matter (g/kg)	7.6 (1.8)	7.6 (1.5)	0.139	.890

Note

Values shown represent means and standard deviations of 10 samples (*df* = 9), *t*‐values, and *p*‐values resulting from Student's *t* tests.

**FIGURE 1:**
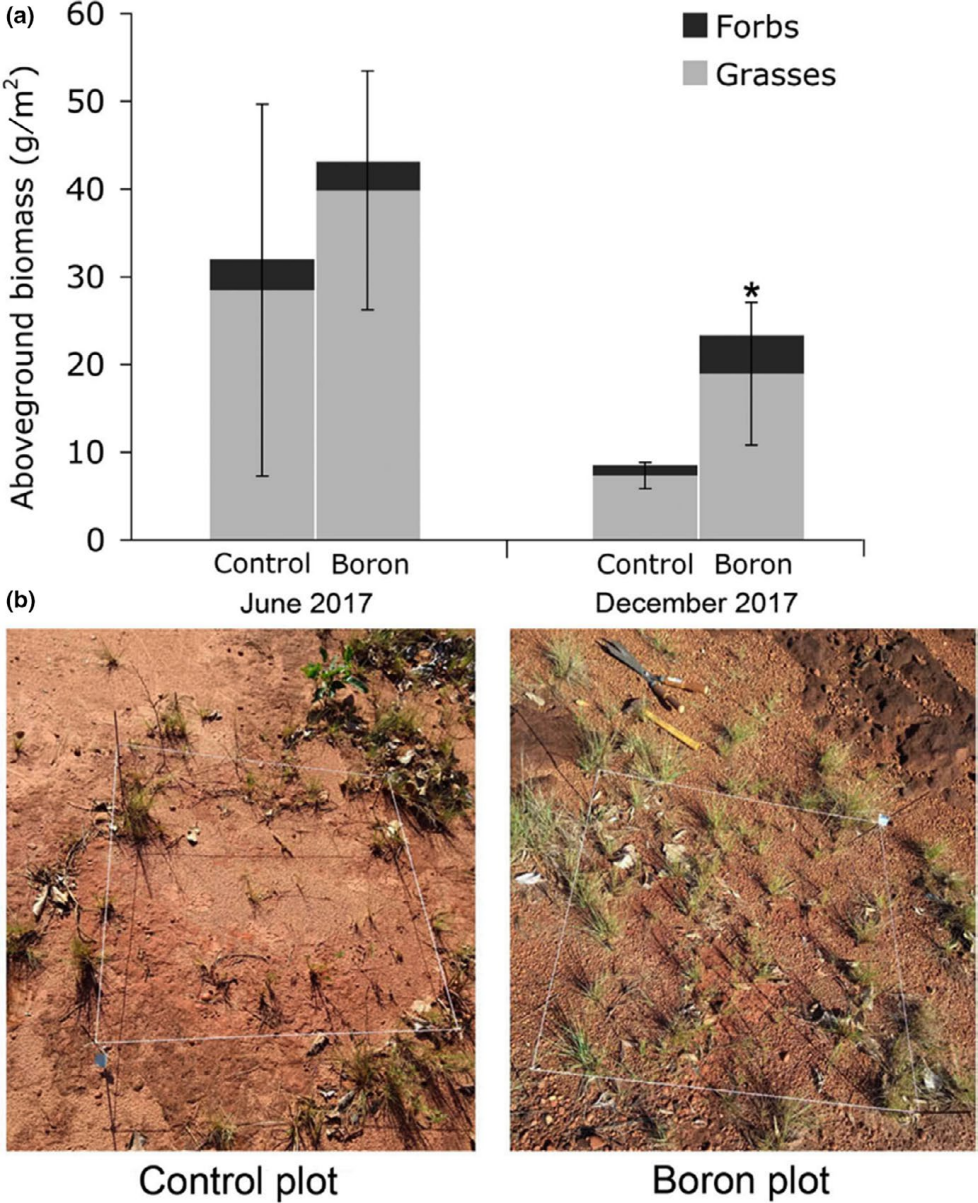
Biomass production of a Cerrado grassland in response to boron addition (0.02 g/m^2^ boron as Borax). (a) Aboveground biomass responses to the addition of boron over two harvest events in a Cerrado grassland. The effect of boron addition on total aboveground biomass was tested using Student's *t* test, *p* < .05. Error bars correspond to the standard deviations of the means of total aboveground biomass (*N* = 10). An asterisk indicates a significant difference for total aboveground biomass between treated and Control plots (for functional groups see Appendix S3). (b) Photographs of one Control plot and one plot treated with boron in December 2017

**FIGURE 2:**
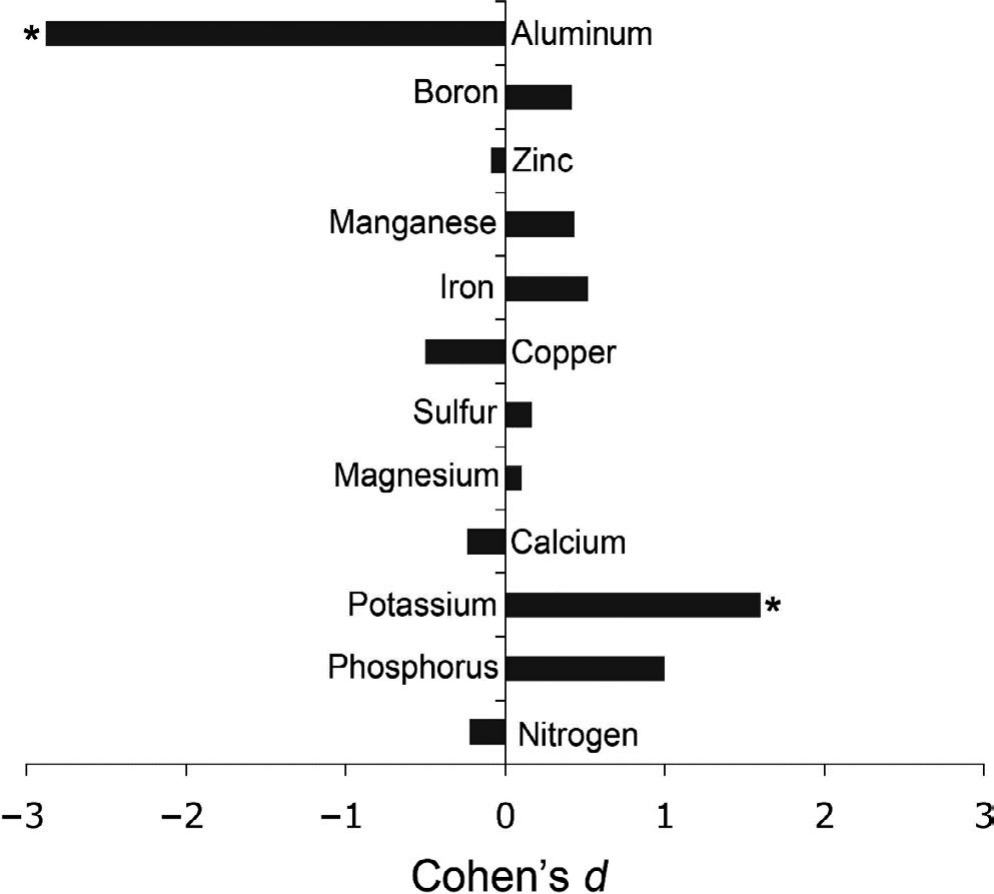
Effect sizes of boron addition (0.02 g/m^2^ boron as Borax) on aboveground nutrient concentrations of grasses (mainly composed of *Hyparrhenia rufa*) in a Cerrado grassland. Positive values show that concentrations in Boron fertilized plots were higher than in the Control plots. Asterisks indicate significant differences (Student's *t* test, *p* < .05) between Boron fertilized and Control plots (*N* = 10). For full statistical results, see Appendix S4

Total aboveground biomass responded positively to B applications in the experimental plots in both harvests (Figure 1; Appendix S3), though not significantly in the first harvest. Lower biomass values were generally detected in the second harvest, but were significantly higher in the B‐treated plots than in Control plots (175% increase, *p = *.008) (Figure 1). Biomass of the plots was mostly composed by vegetative grasses, especially by *Hyparrhenia rufa*, whose response to B addition drove the observed patterns (Appendix S3). There were strong effects (Cohen's *d* > |0.8|) of B applications on tissue concentrations of K and P (positive effects; significantly increased 17% for K (*p = *.007) and tendency of 37% increase for P (*p* = .064)) and highly significant decrease of 211% for Al (*p* < .001) (Figure 2; Appendix S4).

### 3.2 Mesocosm experiment

**FIGURE 3:**
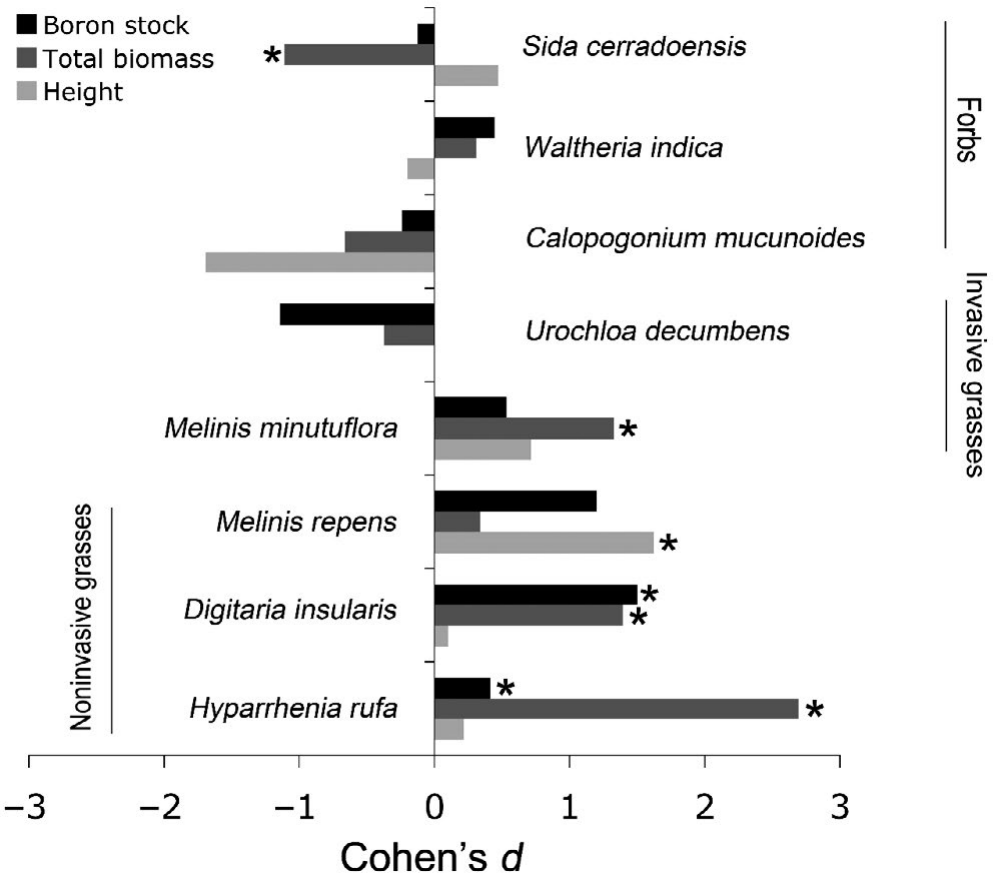
Effect sizes of boron addition (2 mg/kg boron as Borax) on biomass, height, and plant boron stocks of eight Cerrado plants cultivated in a screenhouse. Positive values show that values in Boron fertilized plots were higher than in the Control plots. Asterisks indicate significant differences (Student's *t* test, *p* < .05) between Boron fertilized and Control plots. For full statistical results, see Appendix S5

Boron fertilization promoted a significant increase in soil B comparing unvegetated Control and fertilized plots (0.005–0.4 mg/kg, *p* < .001). Growth of all three noninvasive grasses was increased with B fertilization, with B applications promoting biomass of *Hyparrhenia rufa, Digitaria insularis,* and *Melinis minutiflora* and height of *Melinis repens* (Figure 3; Appendix S5). The first two species also had a significantly higher B stock (biomass x B content), and *M. repens* showed a tendency for this (*p* = .068) (Figure 3). Total biomass of the alien invasive grass *Melinis minutiflora* also significantly increased upon B addition, but the increase in B stock of this species was not significant. *Sida cerradoensis* significantly decreased biomass upon B addition (Appendix S5). Aluminum concentrations in plant tissues of *Hyparrhenia rufa* and *Digitaria insularis* decreased significantly under B fertilization (Appendix S6). The native forbs (*Waltheria indica* and *Calopogonium mucunoides*) and the alien invasive grass *Urochloa decumbens* did not respond to B addition in the mesocosm experiment in terms of biomass, height, or nutrient concentrations (Figure 3; Appendix S5 and Appendix S6). No effect of B addition was detected on root mass ratio (Appendix S5).

## 4 DISCUSSION

Although it is widely known that B is an essential element for plants (Dell & Huang, 1997; Goldbach et al., 2001; Gupta, 1979; Loomis & Durst, 1992; Warington, 1923) and that its addition stimulates growth of some dicotyledonous crop plants, this work is, to our knowledge, the first to demonstrate that B can stimulate growth of monocotyledonous plants and that this element can have implications in preserving grasses in natural, nonagricultural systems.

Silva et al. (2016) reported that the response to B additions may be greater in soils with low organic matter content. This resembles the situation of the degraded experimental area of this study. Indeed, B addition promoted significant increases in total vegetation biomass and biomass of grasses in the studied area in the second harvest. The lack of response in the first harvest might have been caused by the fact that most of the biomass harvested grew before the B addition since vegetation was not clipped before the application of the fertilizer. Moreover, fertilization experiments tend to show clearer patterns of response after a lag phase of low response (Güsewell, Koerselman, & Verhoeven, 2002).

In contrast to previous findings in literature where B addition stimulated growth of forbs (Taylor & MacFie, 1994), in our study B fertilization had the greatest effect on the biomass production of grasses. The grasses in the field experiment area were for 90% composed by the alien but noninvasive grass *Hyparrhenia rufa*. The other 10% was made up by the native Cerrado grasses *Sporobolus indicus* and *Setaria parviflora*. The enhanced growth of these grasses in the field was supported by the results of our mesocosm experiment, where also four grass species (*Hyparrhenia rufa, Digitaria insularis, Melinis repens, and Melinis minutiflora*) increased either their biomass or height upon B addition, whereas forbs did not respond or even decreased in biomass (*Sida cerradoensis*). Noteworthy, the B stocks of *H. rufa, D. insularis,* and *M. repens* changed significantly or tended to change upon B addition, which in combination with the increased biomass and only slightly increased B concentrations is a sign of B limitation; that is, these plants invested the additional acquired B in additional growth. For *M. minutiflora,* B deficiency is less clear because this species did not increase the B stock, and hence growth of this species and also of *Sida cerrodoensis* might have been affected by another factor that was altered by the Borax fertilization.

Higher pH upon B addition could have increased availability of other nutrients than B (cf. Olde Venterink, 2016) However, soil analyses revealed that none of the measured macro‐ or micronutrients had significantly different concentrations after fertilization with Borax and the effect of B on soil pH was clearly not significant (*p* = .333). Moreover, K concentrations in plants increased significantly (*p* = .007) and P concentrations tended to increase as well (*p* = .064). Boron deficiency might have reduced the acquisition of P and K due to the reduced ATPase activity and/or changes in membrane permeability (Pollard, Parr, & Loughman, 1977; Shorrocks, 1990), which can be rapidly restored by the addition of B (Pollard et al., 1977). Both results suggest that plant response to Borax fertilization may be mainly caused by increased B availability rather than to chemical facilitation.

The addition of B induces K absorption because of increased cell membrane permeability, as demonstrated in a laboratory experiment performed by Schon, Novacky, and Blevins (1990). Additions of B to *Helianthus annuus* in a mesocosm experiment (Barman et al., 2014), to *Vicia faba* in a climate chamber (Robertson & Loughman, 1973), and to *Oryza sativa* in the field (Kumar, Arora, & Hundal, 1981) resulted in increased K concentrations in the plants. Although some studies also detected increases in P concentrations in plants such as *Vicia faba* (Robertson & Loughman, 1973), *Gossypium hirsutum* (Ahmed et al., 2011), and *Nicotiana tabacum* (López‐Lefebre et al., 2002) after B fertilization, the reason why foliar P increases after B fertilization is less clear (Rehman et al., 2018) but may be a result of higher cell nuclei metabolism (Shireen et al., 2018).

Boron addition can promote root growth in acidic and high Al soils (Blevins & Lukaszewski, 1998; Lenoble, Blevins, Sharp, et al., 1996; Uluisik, Karakaya, & Koc, 2018) as observed in two grasses in the mesocosm experiment in this study. In high or intermediate pH soils, Al is not available to plants because it is present as Al hydroxides. However, at pH levels below 5 free Al ions increase in the soil solution and might cause plant growth limitation (Kochian, 1995) by impeding root elongation (Ĉiampovorá, 2002). A recent study showed that B deficiency contributes to the Al‐induced inhibition of root elongation by stimulating Al accumulation in the transition zone of the lateral roots of pea plants (*Pisum sativum*) and suggests that plasma membrane‐H^+^‐ATPase is downregulated, resulting in higher root surface pH and therefore decreasing Al accumulation (Li et al., 2018). In alfafa (*Medicago sativa*) and squash (*Cucurbita pepo*), B addition alleviated Al toxicity (Lenoble, Blevins, Sharp, et al., 1996) and promoted positive effects on root and shoot growth. In trifoliate orange (*Poncirus trifoliata*), B addition promoted growth via root protection against Al‐induced oxidative stress (Yan et al., 2019) by stimulating antioxidant enzymes (Riaz et al., 2018). The underlying biochemical and physiological mechanisms regulating B‐induced alleviation of Al toxicity also include cell transport of lipids between membranes (Zhou, Yang, Qi, Guo, & Chen, 2015) and cell wall modification (Li et al., 2017) either due to reduced binding sites for Al (Yu et al., 2009) or by conserving its integrity (Zhou et al., 2015).

Notably, no evidence for beneficial effect of B on plant growth has been reported yet for monocotyledonous plants during vegetative growth phases. Boron addition was specifically tested on the cultivated grass wheat, whereby no alleviation of Al toxicity could be achieved with B addition (Taylor & MacFie, 1994). We could show in this study that B fertilization improved growth of four grasses, had a negative effect on growth of one forb, and had no effect on growth of the other three plant species (two forbs and one alien invasive plant). One of the B‐limited grasses, *Hyparrhenia rufa*, was sensitive to increasing Al by strongly decreasing yield (Brady, 1981), but information on Al tolerance is lacking for the other B‐limited grasses, *Digitaria insularis* and *Melinis repens*. In contrast, some plants that were indifferent toward B addition in the mesocosm experiment were reported to be tolerant to soil Al: that is, the legume *Calopogonium mucunoides* (Meda & Furlani, 2005) and the alien invasive grasses *Melinis minutiflora* and *Urochloa decumbens* (Brady, 1981; Martins, Hay, Walter, Proença, & Vivaldi, 2011). The latter species even increased in yield after Al addition (Brady, 1981). These different plant responses in relation to B fertilization and Al uptake are in accordance with the observed lower concentrations of Al in the field grasses (composed 90% of *Hyparrhenia rufa*) and in two mesocosm grasses under B fertilization and point to the direction that B fertilization might promote plant growth by alleviating Al toxicity in these Cerrado plants, as previously detected Li et al. (2018) in laboratory essays with model plants. Hence, our results are in line with previous studies showing that B deficiency of plants may be associated with Al toxicity, and it is worth to investigate to which extent B deficiency can also occur under soil conditions where Al toxicity is less likely to be an important factor for plant growth (basic to alkaline soils with lower Al availability).

Increased growth of noninvasive grasses upon B addition in the Cerrado agrees with the findings of Lannes et al. (2016), who found that noninvasive grasses were limited by a combination of cations and micronutrients. Non‐native invasive grasses, however, are limited by plant‐available P and have strategies to overcome this limitation by exploiting the strategies of native plants that increase P availability (Lannes, 2012), whereas native forbs in Cerrado grasslands were not limited by nutrients in the Lannes et al. (2016) study. These results suggest that, among Cerrado grasses, the invasion potential seems to be related to the kind of nutrient limitation; that is, plants that respond to P fertilization or use other strategies to obtain P increase their chances of becoming successful invaders in this Al‐rich biome, contrasting to those who are limited by a different nutrient like B. We note, however, that this study was performed in a degraded area in the Cerrado with plants growing in an area with exposed subsoil hardly containing Cerrado native grasses, and whether same patterns are observed in more preserved areas still deserves investigation. Understanding the real nature of nutrient limitation for different functional groups in degraded areas may help managers to consider appropriate approaches for restoring herbaceous Cerrado communities, especially concerning the management of alien invasive grasses.

## CONFLICT OF INTEREST

The authors have no competing interests to report.

## AUTHOR CONTRIBUTION

**Luciola Santos Lannes:** Conceptualization (lead); Data curation (lead); Formal analysis (supporting); Funding acquisition (lead); Investigation (lead); Methodology (lead); Project administration (lead); Resources (lead); Software (supporting); Supervision (lead); Validation (lead); Visualization (lead); Writing‐original draft (supporting); Writing‐review & editing (lead). **Harry Olde Venterink:** Resources (equal); Writing‐review & editing (equal). **Matheus Roberto Leite:** Data curation (equal); Formal analysis (equal); Investigation (equal); Methodology (equal); Validation (equal); Writing‐original draft (equal). **Jaqueline Nascimento Silva:** Data curation (equal); Formal analysis (equal); Investigation (equal); Validation (equal); Writing‐original draft (equal). **Martina Oberhofer:** Writing‐review & editing (equal).

## Supporting information

Appendix S1‐S6

## Data Availability

All data that support the findings of this study will be openly available at Dryad Digital Repository: Lannes, Olde Venterink, Leite, Silva, and Oberhofer (2020), Dryad, Dataset, https://doi.org/10.5061/dryad.4b8gtht93.
