# Supplementary material for: Boron application increases growth of Brazilian Cerrado grasses
Source: Ecol Evol. 2020 May 29;10(13):6364–72. doi: 10.1002/ece3.6367 (PMC7381560; doi:10.1002/ece3.6367)
Supplement: Supplementary file 1 — Appendix S1‐S6 [file ECE3-10-6364-s001.docx]

**Appendix 1** – Description of the methods used to determine foliar nutrients (Malavolta et al. 1997).

| **Plant attribute** | **Extraction method** | **Analytical determination** | **Equipment description** |
| --- | --- | --- | --- |
| Boron | Combustion at 550 °C for 3 hours | Colorimetry due to reaction with Azomethine-H. Spectrophotometer 420 nm | Biospectro SP 220 |
| Aluminium | Nitric-perchloric digestion conc. 2:1 v.v. | Colorimetry due to complexation with aluminon. Spectrophotometer 520 nm | Biospectro SP 220 |
| Nitrogen | Kjeldahl | Titration with 0.05 N HCl | pH meter Metrohm Herisau E250 with Mettler Toledo electrode (3 M KCl) |
| Phosphorus | Nitric-perchloric digestion conc. 2:1 v.v. | Colorimetry due to reaction with ammonium molybdate and metavanadate. Spectrophotometer 420 nm | Biospectro SP 220 |
| Potassium | Nitric-perchloric digestion conc. 2:1 v.v. | Atomic absorption | Varian Spectraa, 55B |
| Calcium | Nitric-perchloric digestion conc. 2:1 v.v. | Atomic absorption | Varian Spectraa, 55B |
| Magnesium | Nitric-perchloric digestion conc. 2:1 v.v. | Atomic absorption | Varian Spectraa, 55B |
| Sulfur | Nitric-perchloric digestion conc. 2:1 v.v. | Turbidimetry due to reaction of S-SO_4_^2-^ with BaCl_2_.H_2_O PA. Spectrophotometer 420 nm. | Biospectro SP 220 |
| Copper | Nitric-perchloric digestion conc. 2:1 v.v. | Atomic absorption | Varian Spectraa, 55B |
| Iron | Nitric-perchloric digestion conc. 2:1 v.v. | Atomic absorption | Varian Spectraa, 55B |
| Manganese | Nitric-perchloric digestion conc. 2:1 v.v. | Atomic absorption | Varian Spectraa, 55B |
| Zinc | Nitric-perchloric digestion conc. 2:1 v.v. | Atomic absorption | Varian Spectraa, 55B |

**Appendix 2** – Description of the methods used to determine soil nutrients (Raij et al. 2001).

| **Soil attribute** | **Extraction method** | **Analytical determination** | **Equipment description** |
| --- | --- | --- | --- |
| Boron | Heating in microwave at 490 W for 5 min in a solution of barium chloride 6 mM (1.25 g L^-1^) (*) | Colorimetry due to reaction with Azomethine-H. Spectrophotometer 420 nm | Varian 50 Probe |
| Aluminium | Shaking at 20 rpm for 5 min in 1 M KCl | Titration with NaOH 0.025 mol L^-1^ with phenolphthalein as indicator | pH meter Metrohm Herisau E250 with Mettler Toledo electrode (3 M KCl) |
| Calcium | Shaking at 220 rpm for 16 hours with ion exchange resin (Amberlite IRA 400) and then washed with 0.8 M NH_4_Cl and 0.2 M HCl | Atomic absorption | Varian Spectraa, 55B |
| Cupper | Shaking at 220 rpm for 2 hours in 0.005 M DTPA + 0.1 M triethalonamine + 0.01 M CaCl_2_ solution at pH 7.3 | Atomic absorption | Varian Spectraa, 55B |
| Iron | Shaking at 220 rpm for 2 hours in 0.005 M DTPA + 0.1 M triethalonamine + 0.01 M CaCl_2_ solution at pH 7.3 | Atomic absorption | Varian Spectraa, 55B |
| Magnesium | Shaking at 220 rpm for 16 hours with ion exchange resin (Amberlite IRA 400) and then washed with 0.8 M NH_4_Cl + 0.2 M HCl | Atomic absorption | Varian Spectraa, 55B |
| Manganese | Shaking at 220 rpm for 2 hours in 0.005 M DTPA + 0.1 M triethalonamine + 0.01 M CaCl_2_ solution at pH 7.3 | Atomic absorption | Varian Spectraa, 55B |
| Phosphorus | Shaking at 220 rpm for 16 hours with ion exchange resin (Amberlite IRA 400) and then washed with 0.8 M NH_4_Cl + 0.2 M HCl | Colorimetry due to reaction with molybdenum blue. Spectrophotometer 650 nm | Varian 50 Probe |
| Potassium | Shaking at 220 rpm for 16 hours with ion exchange resin (Amberlite IRA 400) and then washed with 0.8 M NH_4_Cl + 0.2 M HCl | Atomic absorption | Varian Spectraa, 55B |
| Sulfur | Shaking with activated charcoal and 0.01 M Ca(H_2_PO_4_)_2_ | Turbidimetry due to reaction of S-SO_4_^2-^ with BaCl_2_.H_2_O PA. Spectrophotometer 420 nm | Varian 50 Probe |
| Zinc | Shaking at 220 rpm for 2 hours in 0.005 M DTPA + 0.1 M triethalonamine + 0.01 M CaCl_2_ solution at pH 7.3 | Atomic absorption | Varian Spectraa, 55B |
| pH | Water-soil suspension 10 grams air-dry soil < 2 mm with 50 mL deionised water | pH meter | pH meter Metrohm Herisau E250 with Mettler Toledo electrode (3 M KCl) |
| Organic matter | Shaking at 180 rpm for 10 min with 0.667 M sodium dichromate (Na_2_Cr_2_O_7_.2H_2_O) and 5 M sulphuric acid | Colorimetry due to reaction with sodium dichromate. Spectrophotometer 650 nm | Varian 50 Probe |

(*)Method from Abreu CA, Abreu MF, Raij B, Bataglia OC & Andrade JC (1994) Extraction of boron from soil by microwave heating for ICP-AES determination. Communications in Soil Science and Plant Analysis, New York, v.25, p.3321-3333. (Adapted from Berger KC & Truog E (1939) Boron determination in soils and plants. *Industrial and Engineering Chemistry Analytical Edition*. Washington, v.11, p.540-545). This method yields results comparable to the traditional hot water extraction method (Sah & Brown 1997; Crusciol et al. 2018).

**Appendix 3** – Aboveground biomass of grasses and forbs in plots receiving 0.02 g.m^-2^ boron as Borax and unfertilized control plots June and December 2017. Means and standard deviations of the means are shown (N=10). Asterisks indicate significant differences between treatment and control plots in the period (Student t-test, P<0.05).

|  | **Grasses** (g m^-2^) | **Forbs** (g m^-2^) |
| --- | --- | --- |
| **June** |  |  |
| Control | 28.47 (20.4) | 3.50 (3.10) |
| Boron | 39.86 (9.16) | 3.26 (3.41) |
| t-value | 1.610 | 0.164 |
| P-value | 0.124 | 0.871 |
|  |  |  |
| **December** |  |  |
| Control | 7.35(6.29) | 1.15(0.90) |
| Boron | 18.94(8.49)* | 4.40(2.24)* |
| t-value | 5.768 | 4.257 |
| P-value | <0.001 | <0.001 |

**Appendix 4 –** Means and standard deviations of nutrient concentrations in aboveground tissues of the vegetation (mainly composed by *Hyparrhenia rufa*) in unfertilized and boron-fertilized plots (0.02 g.m^-2^ boron as Borax) in a Cerrado area. Asterisks indicate significant differences at P <0.05 in Student's t-test, N=10. This data was used to build the Cohen's diagram shown in Figure 2.

| **Element** | **Control** | **+Boron** | **t-value** | **P-value** |
| --- | --- | --- | --- | --- |
| Nitrogen (g.kg^-1^) | 8.9(1.2) | 8.7(0.6) | 0.511 | 0.615 |
| Phosphorus (g.kg^-1^) | 0.8(0.3) | 1.1(0.3) | 1.937 | 0.064 |
| Potassium (g.kg^-1^) | 2.3(0.2) | 2.7(0.3)* | 2.997 | 0.007 |
| Calcium (g.kg^-1^) | 1.1(1.0) | 0.9(0.7) | 0.518 | 0.610 |
| Magnesium (g.kg^-1^) | 0.8(0.8) | 0.9(1.1) | 0.232 | 0.819 |
| Sulfur (g.kg^-1^) | 1.4(0.3) | 1.5(0.9) | 0.333 | 0.742 |
| Copper (mg.kg^-1^) | 10(5) | 8(3) | 1.084 | 0.292 |
| Iron (mg.kg^-1^) | 1060(451) | 1324(634) | 1.037 | 0.297 |
| Manganese (mg.kg^-1^) | 139(31) | 161(70) | 0.908 | 0.375 |
| Zinc (mg.kg^-1^) | 15(10) | 14(13) | 0.193 | 0.849 |
| Boron (mg.kg^-1^) | 58(21) | 66(17) | 0.936 | 0.361 |
| Aluminium (g.kg^-1^) | 261(92)* | 84(31) | 5.765 | <0.001 |

**Appendix 5** – Effect of boron addition (2 mg kg^-1^ boron as Borax) on biomass (g per mesocosm), root mass ratio (mg per mg), height (cm per mesocosm) and boron stock (mg per mesocosm) of eight Cerrado species. Values shown are means and standard deviations in parentheses, degrees of freedom, t and P values resulting from Student t-tests.

|  | **Control** | **+Boron** | **df** | **t-value** | **P-value** |
| --- | --- | --- | --- | --- | --- |
| ***Hyparrhenia rufa*** |  |  |  |  |  |
| Aerial biomass | 0.53(0.21) | 0.88(0.12) | 9 | 3.210 | 0.014 |
| Root biomass | 0.39(0.15) | 0.66(0.16) | 9 | 2.830 | 0.022 |
| Total biomass | 0.92(0.3) | 1.54(0.16) | 9 | 5.436 | <0.001 |
| Root mass ratio | 0.75(0.26) | 0.77(0.24) | 9 | 0.115 | 0.910 |
| Height | 99(21) | 106(43) | 9 | 0.460 | 0.790 |
| Boron stock | 0.05(0.31) | 0.12(0.03) | 4 | 2.979 | 0.018 |
|  |  |  |  |  |  |
| ***Digitaria insularis*** |  |  |  |  |  |
| Aerial biomass | 0.82(0.40) | 1.18(0.39) | 9 | 2.640 | 0.029 |
| Root biomass | 0.31(0.12) | 0.55(0.15) | 9 | 2.650 | 0.029 |
| Total biomass | 1.14(0.48) | 1.74(0.38) | 9 | 3.129 | 0.006 |
| Root mass ratio | 0.29(0.09) | 0.33(0.11) | 9 | 0.793 | 0.438 |
| Height | 85(57) | 89(22) | 9 | 0.150 | 0.887 |
| Boron stock | 0.08(0.04) | 0.14(0.04) | 4 | 2.145 | 0.042 |
|  |  |  |  |  |  |
| ***Melinis repens*** |  |  |  |  |  |
| Aerial biomass | 1.18(0.41) | 1.14(0.22) | 9 | 0.180 | 0.861 |
| Root biomass | 0.54(0.16) | 0.52(0.10) | 9 | 0.164 | 0.873 |
| Total biomass | 1.47(0.68) | 1.66(0.44) | 9 | 0.731 | 0.463 |
| Root mass ratio | 0.40(0.09) | 0.33(0.11) | 9 | 1.643 | 0.118 |
| Height | 62(10) | 75(6) | 8 | 2.550 | 0.034 |
| Boron stock | 0.06(0.03) | 0.12(0.07) | 4 | 2.108 | 0.068 |
|  |  |  |  |  |  |
| ***Melinis minutiflora*** |  |  |  |  |  |
| Aerial biomass | 2.24(0.32) | 2.83(0.52) | 9 | 2.120 | 0.067 |
| Root biomass | 0.92(0.41) | 1.18(0.12) | 9 | 1.360 | 0.210 |
| Total biomass | 3.16(0.66) | 4.01(0.62) | 9 | 2.954 | 0.008 |
| Root mass ratio | 0.27(0.11) | 0.29(0.04) | 9 | 0.644 | 0.528 |
| Height | 62(6) | 67(8) | 9 | 1.020 | 0.337 |
| Boron stock | 0.17(0.06) | 0.21(0.09) | 4 | 0.547 | 0.434 |
|  |  |  |  |  |  |
| ***Urochloa decumbens*** |  |  |  |  |  |
| Aerial biomass | 0.84(0.18) | 0.81(0.19) | 9 | 0.030 | 0.868 |
| Root biomass | 0.45(0.13) | 0.39(0.14) | 9 | 0.689 | 0.509 |
| Total biomass | 1.29(0.27) | 1.19(0.27) | 9 | 0.817 | 0.424 |
| Root mass ratio | 0.35(0.07) | 0.32(0.09) | 9 | 0.663 | 0.516 |
| Height | 50(5) | 50(6) | 9 | 0.080 | 0.787 |
| Boron stock | 0.12(0.05) | 0.08(0.02) | 4 | 0.716 | 0.369 |
|  |  |  |  |  |  |
| ***Calopogonium mucunoides*** |  |  |  |  |  |
| Aerial biomass | 0.92(0.65) | 0.72(0.21) | 6 | 0.600 | 0.574 |
| Root biomass | 0.53(0.72) | 0.19(0.08) | 6 | 0.952 | 0.384 |
| Total biomass | 1.45(1.35) | 0.91(0.28) | 6 | 0.965 | 0.357 |
| Root mass ratio | 0.25(0.17) | 0.21(0.05) | 6 | 0.488 | 0.636 |
| Height | 57(4) | 46(9) | 5 | 1.870 | 0.135 |
| Boron stock | 0.07(0.06) | 0.04(0.19) | 4 | 0.494 | 0.257 |
|  |  |  |  |  |  |
| ***Waltheria indica*** |  |  |  |  |  |
| Aerial biomass | 1.08(0.17) | 1.16(0.57) | 9 | 0.270 | 0.792 |
| Root biomass | 0.17(0.07) | 0.24(0.13) | 9 | 0.790 | 0.452 |
| Total biomass | 1.26(0.19) | 1.39(0.65) | 9 | 0.620 | 0.543 |
| Root mass ratio | 0.14(0.04) | 0.18(0.09) | 9 | 1.247 | 0.228 |
| Height | 30(3) | 29(7) | 9 | 0.520 | 0.613 |
| Boron stock | 0.09(0.03) | 0.11(0.06) | 4 | 0.461 | 0.507 |
|  |  |  |  |  |  |
| ***Sida cerradoensis*** |  |  |  |  |  |
| Aerial biomass | 0.38(0.25) | 0.16(0.04) | 9 | 2.770 | 0.140 |
| Root biomass | 0.26(0.20) | 0.17(0.14) | 9 | 0.610 | 0.548 |
| Total biomass | 0.63(0.40) | 0.33(0.14) | 9 | 2.233 | 0.038 |
| Root mass ratio | 0.43(0.14) | 0.45(0.21) | 9 | 0.366 | 0.719 |
| Height | 18(2) | 22(15) | 9 | 0.510 | 0.497 |
| Boron stock | 0.03(0.02) | 0.02(0.14) | 4 | 0.542 | 0.481 |
|  |  |  |  |  |  |

**Appendix 6** – Effect of boron addition (2 mg kg^-1^ boron as Borax) on tissue nutrients of eight Cerrado species. Values shown are means and standard deviations in parentheses, and P values resulting from Student t-tests. N=5.

|  | ***Hyparrhenia rufa*** | | | ***Digitaria insularis*** | | | ***Melinis repens*** | | | ***Melinis minutiflora*** | | |
| --- | --- | --- | --- | --- | --- | --- | --- | --- | --- | --- | --- | --- |
| **Plant attribute** | **Control** | **Boron** | **P-value** | **Control** | **Boron** | **P-value** | **Control** | **Boron** | **P-value** | **Control** | **Boron** | **P-value** |
| Boron (mg.kg^-1^) | 63(18) | 74(13) | 0.268 | 98(41) | 87(15) | 0.569 | 52(20) | 61(20) | 0.520 | 57(14) | 53(14) | 0.666 |
| Aluminium (g.kg^-1^) | 174(71) | 47(11) | 0.001 | 196(54) | 112(21) | 0.012 | 84(23) | 75(17) | 0.504 | 66(23) | 61(19) | 0.720 |
| Nitrogen (g.kg^-1^) | 7.5(3.1) | 8.2(1.1) | 0.430 | 11.2(1.5) | 10.0(2.8) | 0.587 | 14(1) | 14(5) | 0.716 | 7.6(2.0) | 6.2(1.4) | 0.254 |
| Phosphorus (g.kg^-1^) | 0.9(0.4) | 1.0(0.2) | 0.848 | 1.5(1.1) | 1.4(0.8) | 0.975 | 2.0(1.1) | 1.8(0.2) | 0.661 | 0.8(0.2) | 0.9(0.1) | 0.700 |
| Potassium (g.kg^-1^) | 2.9(0.3) | 2.7(0.6) | 0.491 | 5.9(1.4) | 6.4(1.9) | 0.607 | 1.2(0.4) | 1.4(0.1) | 0.461 | 1.8(0.3) | 1.6(0.4) | 0.395 |
| Calcium (g.kg^-1^) | 1.2(0.6) | 0.9(0.5) | 0.387 | 3.6(0.7) | 2.8(0.9) | 0.183 | 0.3(0.1) | 0.5(0.0) | 0.079 | 0.7(0.2) | 0.8(0.2) | 0.687 |
| Magnesium (g.kg^-1^) | 1.0(0.7) | 0.7(0.2) | 0.260 | 2.0(0.4) | 1.9(0.7) | 0.956 | 0.6(0.2) | 0.4(0.4) | 0.455 | 1.8(0.9) | 1.7(1.3) | 0.936 |
| Sulfur (g.kg^-1^) | 1.2(0.3) | 1.5(0.4) | 0.250 | 0.8(0.7) | 1.4(0.2) | 0.106 | 0.9(0.5) | 0.5(0.2) | 0.112 | 1.3(0.5) | 1.2(0.4) | 0.944 |
| Copper (mg.kg^-1^) | 10(3) | 10(2) | 0.667 | 6(3) | 8(1) | 0.162 | 14(2) | 13(2) | 0.384 | 10(2) | 10(2) | 0.849 |
| Iron (g.kg^-1^) | 1.2(0.1) | 1.2(0.6) | 0.942 | 0.7(0.2) | 0.6(0.2) | 0.309 | 1.0(0.3) | 1.2(0.4) | 0.359 | 1.3(0.5) | 1.3(0.3) | 0.825 |
| Manganese (mg.kg^-1^) | 147(29) | 178(37) | 0.176 | 125(52) | 116(24) | 0.703 | 166(58) | 204(63) | 0.358 | 153(29) | 142(43) | 0.648 |
| Zinc (mg.kg^-1^) | 15(4) | 13(5) | 0.599 | 65(14) | 74(45) | 0.673 | 57(24) | 69(15) | 0.368 | 46(19) | 55(24) | 0.559 |

|  | ***Urochloa decumbens*** | | | ***Sida cerradoensis*** | | | ***Waltheria indica*** | | | ***Calopogonium mucunoides*** | | |
| --- | --- | --- | --- | --- | --- | --- | --- | --- | --- | --- | --- | --- |
| **Plant attribute** | **Control** | **Boron** | **P-value** | **Control** | **Boron** | **P-value** | **Control** | **Boron** | **P-value** | **Control** | **Boron** | **P-value** |
| Boron (mg.kg^-1^) | 71(22) | 66(23) | 0.744 | 49(25) | 49(26) | 0.991 | 73(20) | 76(16) | 0.744 | 51(24) | 50(26) | 0.952 |
| Aluminium (g.kg^-1^) | 56(26) | 63(24) | 0.692 | 67(22) | 61(20) | 0.674 | 50(26) | 52(31) | 0.890 | 64(12) | 64(15) | 0.946 |
| Nitrogen (g.kg^-1^) | 4.6(1.5) | 5.2(1.5) | 0.545 | 5.6(2.5) | 5.6(2.1) | 1.000 | 5.2(2.8) | 4.8(0.8) | 0.766 | 16.4(4.1) | 17.2(10.0) | 0.873 |
| Phosphorus (g.kg^-1^) | 1.0(0.2) | 0.9(0.2) | 0.799 | 1.0(0.3) | 0.9(0.2) | 0.410 | 1.0(0.1) | 1.1(0.3) | 0.497 | 1.0(0.2) | 0.9(0.3) | 0.425 |
| Potassium (g.kg^-1^) | 1.5(0.9) | 1.3(0.5) | 0.641 | 1.8(1.2) | 1.7(0.6) | 0.900 | 2.0(0.8) | 1.6(0.8) | 0.458 | 1.4(0.7) | 1.3(0.3) | 0.787 |
| Calcium (g.kg^-1^) | 2.6(1.3) | 2.2(0.3) | 0.605 | 2.4(1.3) | 2.9(2.3) | 0.730 | 4.0(1.7) | 4.3(0.9) | 0.738 | 1.1(0.3) | 1.0(0.1) | 0.371 |
| Magnesium (g.kg^-1^) | 1.2(0.4) | 1.0(0.1) | 0.450 | 1.2(0.6) | 1.0(0.3) | 0.688 | 2.2(0.9) | 2.4(1.3) | 0.798 | 1.4(0.7) | 1.5(0.9) | 0.814 |
| Sulfur (g.kg^-1^) | 1.6(0.8) | 1.4(1.1) | 0.800 | 0.7(0.7) | 0.7(0.3) | 0.958 | 1.0(0.15) | 0.9(0.2) | 0.455 | 1.6(1.0) | 1.6(1.0) | 1.000 |
| Copper (mg.kg^-1^) | 15(14) | 13(2) | 0.786 | 12(3) | 12(6) | 0.794 | 10(1) | 9.4(2.1) | 0.586 | 11(2) | 10(2) | 0.384 |
| Iron (g.kg^-1^) | 1.2(0.7) | 1.0(0.3) | 0.517 | 1.1(0.5) | 1.4(0.5) | 0.386 | 1.4(0.5) | 1.4(1.0) | 0.939 | 1.0(0.2) | 0.9(0.2) | 0.578 |
| Manganese (mg.kg^-1^) | 157(17) | 137(31) | 0.240 | 153(39) | 141(58) | 0.698 | 150(32) | 146(27) | 0.837 | 126(35) | 118(36) | 0.719 |
| Zinc (mg.kg^-1^) | 57(28) | 55(32) | 0.919 | 55(26) | 51(30) | 0.82 | 46(23) | 37(28) | 0.594 | 64(26) | 60(29) | 0.824 |
